# The Impact of Drug Properties and Severity of Obesity on Renal Drug Clearance Through Glomerular Filtration and Active Tubular Secretion: A Systematic Analysis Using PBPK Modeling

**DOI:** 10.1007/s11095-025-03885-5

**Published:** 2025-06-27

**Authors:** Tan Zhang, Elisa A. M. Calvier, Elke H. J. Krekels, Catherijne A. J. Knibbe

**Affiliations:** 1 Division of Systems Pharmacology and Pharmacy, Leiden Academic Centre for Drug Research, Leiden University, Leiden, The Netherlands; 2 Certara Inc, Princeton, NJ USA; 3 Pharmacokinetics-Dynamics and Metabolism, Translational Medicine and Early Development, Sanofi R&D, Montpellier, France; 4 Department of Clinical Pharmacy, St. Antonius Hospital, Nieuwegein, The Netherlands

**Keywords:** allometric scaling, clearance scaling, obesity, PBPK modeling, renal transporter activity

## Abstract

**Objective:**

The influence of obesity on renal drug clearance (CLr) remains difficult to predict. This study quantifies obesity-related alterations in CLr for drugs eliminated via glomerular filtration (GF/CL_GF_) and active tubular secretion (ATS/CL_ATS_) and assesses the systematic accuracy of dosing based on allometric scaling with an exponent of 0.75 or flat dosing (exponent of 0).

**Methods:**

A physiologically-based pharmacokinetic (PBPK) approach was used to simulate CL_GF_ and CL_ATS_ for 11,520 hypothetical drugs in typical subjects with body mass index (BMI) between 20 and 60. Correlations between changes in CL_GF_ and CL_ATS_ and subject or drug properties were investigated. Moreover, for each drug, CLr values scaled to individuals with obesity from CLr values in normal-weight individuals were compared to PBPK predictions of CLr. Systematic scaling accuracy was defined as the prediction error being less than ± 30% for all drugs.

**Results:**

CLr through GF and ATS increased with BMI, albeit to different extents, depending on drug properties. When BMI was below 30 kg/m^2^ and transporter activity remained unchanged, the CLr between subjects of normal weight and with overweight or obesity differed less than 30% and both scaling methods were systematically accurate. For individuals with higher BMI, drug properties need to be taken into account when defining scenarios of systematic scaling accuracy.

**Conclusion:**

In individuals with a BMI above 30 kg/m^2^, neither 0.75 allometric scaling nor no scaling (flat dosing) is systematically accurate for renally cleared drugs. Strategies are provided to define systematic scaling accuracy *a priori*, based on subject and drug properties.

**Supplementary Information:**

The online version contains supplementary material available at 10.1007/s11095-025-03885-5.

## Introduction

Renal drug clearance (CLr) is influenced by various processes including, amongst others, glomerular filtration (GF) and active tubular secretion (ATS). Obesity is a growing global health concern. Although its influence on CLr has been studied for individual drugs, a systematic analysis quantifying its impact on CLr in relation to different drug properties is lacking [1, 2]. Obesity can, for instance, lead to a higher glomerular filtration rate, but from clinical studies, it appears that this does not consistently result in a corresponding rise in CLr, with studies showing enhanced CLr for some drugs in obese individuals, and unaltered CLr for others [2, 3]. This could stem from alterations in ATS counteracting the impact of GF alterations. Although obesity is thought to influence subject-related parameters such as transporter activity and, in turn, affect substance elimination via ATS [2, 4], information on specific obesity-induced changes in transporter activity is scarce, due to limited studies on this topic. As a result, the impact of obesity on ATS remains unclear. This knowledge gap makes it challenging to understand and predict obesity-related changes in the underlying mechanism of obesity influencing CLr.

Meanwhile, simple clearance scaling methods that provide a convenient way to predict changes in CLr in individuals with obesity in those cases where no data are available from clinical studies, are sought after [2]. This is because changes in CL can often be directly translated into required changes of the maintenance dose. When dosing patients with obesity, two strategies are most commonly used: flat dosing, in which the same dose is administered to individuals with both normal weight and obesity, or a bodyweight-based exponential scaling strategy with an exponent of 0.75 (AS0.75) [5]. So far, the accuracy of these scaling methods for renally cleared drugs in the obese population has not been systematically evaluated.

In order to predict the obesity-induced changes in pharmacokinetics (PK) and the necessary dose adjustments in those cases where there is no evidence from clinical studies or data including novel drugs, physiologically based PK (PBPK) principles can be used. With PBPK, CL changes can be derived for drugs with various drug properties from obesity-related physiological changes. However, applying a full PBPK model to make predictions for each drug requires a substantial amount of quantitative information on drug properties, some of which may be still unknown. Instead, a PBPK simulation workflow as developed previously by Calvier *et al.* [6–8] for the pediatric population, can be used to generate CL values for drugs of different properties, upon which a systematic evaluation of the accuracy of scaling methods can be performed. Such a method enables the definition of scenarios that lead to accurate weight-based scaling based on subject characteristics and drug properties. This allows for the definition of the minimal information required to select accurate clearance scaling methods.

This PBPK-based simulation study quantifies obesity-related alterations in CLr for drugs eliminated via GF and ATS across the entire drug-parameter space. Additionally, it explores the impact of potential obesity-induced changes in ATS on the total CLr for these drugs, considering hypothetical relative changes in transporter activity (rTA) with obesity. Finally, in order to provide *a priori* guidance on selecting the most appropriate scaling method for subjects with overweight or obesity, scenarios are defined where commonly used scaling methods, like allometric scaling with an exponent of 0.75 or flat dosing (exponent of 0), lead to systematically accurate scaled CLr values.

## Materials and Methods

### Renal Clearance Simulation for Normal-weight and Obese Subjects

A PBPK-based simulation workflow was used to perform simulations in R (version 4.0.3) under R studio (version 1.1.43) for drugs eliminated via GF and ATS. Six BMI categories were investigated using typical subjects. Typical subjects incorporated in this simulation include a normal-weight subject (BMI 20 kg/m^2^), an overweight subject (BMI 25 kg/m^2^), and four (morbidly) obese subjects with BMI of 30, 40, 50, and 60 kg/m^2^. The corresponding bodyweight of these subjects was calculated based on a typical height of 1.72 meter, which is the median height value for adults in the NHANES database [9], yielding weights of 59.2, 74.0, 88.8, 118.3, 148.0, and 177.5 kg, respectively.

**Fig. 1:**
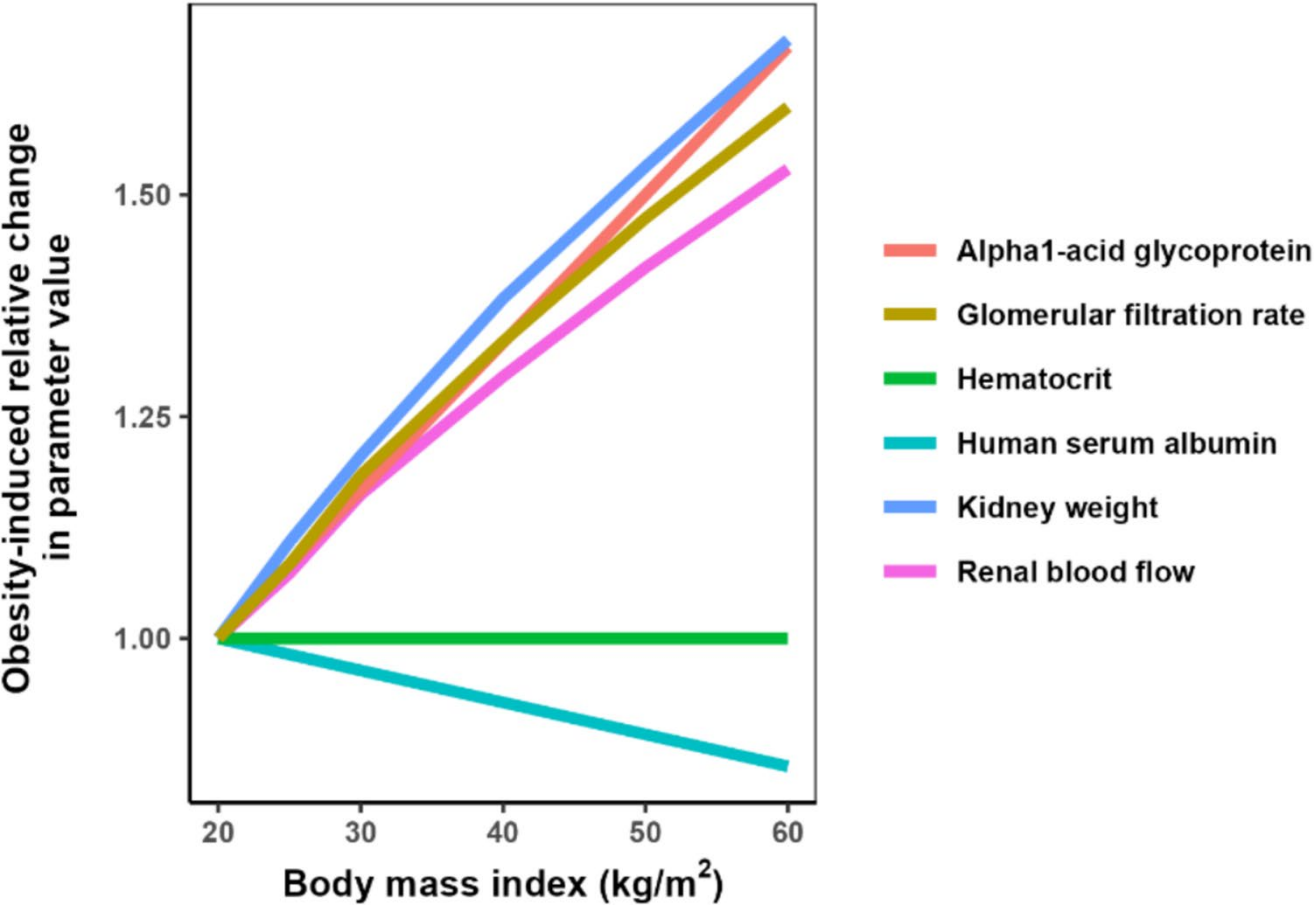
Obesity-induced changes in system-specific parameters relative to normal-weight subjects (based on Berton *et al.*)

The subject-specific parameters involved in the CLr simulation included renal blood flow (Qr), kidney weight, plasma concentrations of human serum albumin (HSA) or alpha-1 acid glycoprotein (AAG), hematocrit, glomerular filtration rate (GFR), and the number of proximal tubule cells per gram kidney (PTCPGK). The value of PTCPGK was 99.4, obtained from SimCYP Simulator® (version 22) and in the absence of information suggesting otherwise, this was assumed to remain unaltered with BMI. The values of the other parameters were calculated based on the equations reported by Berton *et al. *[10]. The equations and the corresponding values of subject-specific parameters for each typical subject are provided in the supplemental materials, and the obesity-induced changes in subject-specific parameters relative to normal-weight subjects are illustrated in Fig. 1.

To explore obesity-induced changes in CLr across the entire drug-parameter space, a multiparameter sensitivity analysis was conducted, encompassing 11,520 distinct parameter combinations, each representing a hypothetical drug undergoing CLr. Specifically, these hypothetical drugs were characterized by three drug-specific parameters. For each parameter, multiple values were selected within a realistic range of values:

1. unbound drug fraction in plasma in normal-weight subjects (fu_normal weight_) with values of 5%, 25%, 50%, 75%, 95%, and 100%. Drugs were assumed to exclusively bind to either HSA or AAG;
2. blood-to-plasma partition coefficient (K_p_) with values of 0.35, 1, 2, 3, 4, 5, 10, 20, 30, and 40 to indicate different degrees of drug diffusion into red blood cells [11, 12];
3. intrinsic active transporter-mediated secretion clearance (CL_int_ATS_) with values from 2 to 500 µl·min^−1^mg protein^−1^ with 32 different equidistant values, to reflect the drug affinity to the tubular secretion transporters together with the abundances of the transporters.

As obesity-induced alterations in the activity of renal transporters remain largely unknown, due to limited clinical data and a lack of comprehensive literature [13, 14], obesity-induced changes in transporter activity are studied as hypothetical percentages of transporter activity compared to normal-weight individuals, referred to as relative transporter activity (rTA). For each BMI category, percentage changes between 20 and 250% were studied (i.e., 20%, 50%, 100%, 150%, 200%, 250%), with an rTA value of 100% indicating no change in transporter activity in individuals with obesity compared to normal-weight individuals. This range was derived from existing literature on changes in kidney transporter activity in obese or diabetic mice [15–17]; when clinical information on specific transporters becomes available it will be possible to select the most relevant scenarios. Although it is unlikely that large changes in rTA occur at relatively low degrees of overweight or obesity, it is unknown what ranges are relevant for each BMI category and therefore the entire rTA range was tested for each BMI category.

Parameters that are both drug-specific and subject-specific, including fu, blood-to-plasma ratio (BP), and the total intrinsic secretion clearance (CL_int_sec_), were calculated based on the subject- and drug-specific parameters as described in the supplemental materials. The fu in subjects with obesity was calculated based on fu_normal weight_ and the plasma concentrations of the drug binding plasma proteins, being either HSA or AAG, and was negatively correlated with the plasma concentration of the drug binding protein [18, 19]. The affinity of drugs to these plasma proteins was assumed to remain constant with varying BMI. BP was calculated based on hematocrit, fu, and K_p_, where hematocrit was influenced only by sex according to Berton *et al. *[10]*,* and K_p_ was assumed to be constant across the BMI range. CL_int_sec_ was calculated as the product of CL_int_ATS_, kidney weight, PTCPGK, and rTA.

With these parameters, CLr was predicted using the PBPK models shown in Eqs. 1–3. The total CLr was assumed to be the sum of the clearance through GF and ATS as outlined in Eq. 1. The calculations for CLr through GF and ATS are defined by Eqs. 2 and 3, respectively.

1 $${CL}_{r}={CL}_{GF}+{CL}_{ATS}$$

2 $${CL}_{GF}=fu\times GFR$$

3 $${CL}_{ATS}=\frac{\left(Qr-GFR\right)\times fu\times CL_{int\_sec}}{Qr+fu\times{\displaystyle\frac{CL_{int\_sec}}{BP}}}$$

### Obesity-related Changes in Renal Clearance

With the PBPK model, CLr was predicted for all 11,520 hypothetical drugs in all six BMI categories. Relative CLr in subjects with overweight or obesity, compared to normal-weight individuals, was calculated. The correlation among relative CLr with subject properties and drug properties was investigated graphically. Besides, the contribution of GF and ATS to CLr was determined according to Eqs. 4 and 5 respectively.

4 $$contribution\;of\;GF\;\left(\boldsymbol\%\right)=\frac{{CL}_{GF}}{CLr}\times100\%$$

5 $$contribution\;of\;ATS\;\left(\boldsymbol\%\right)=\frac{{CL}_{ATS}}{CLr}\times100\%$$

### Systematic Accuracy of CLr Scaling Methods

For each hypothetical drug, CLr was scaled from normal-weight subjects to overweight and obese subjects according to Eq. 6. Values for the exponent of 0 and 0.75 were used to represent CLr scaling that is associated with flat dosing and AS0.75-based dosing, respectively.

6 $${scaled\;CLr}_{obese}={CLr}_{normal\;weight}\times\left(\frac{{BW}_{obese}}{{BW}_{normal\;weight}}\right)^{exponent}$$

The prediction error (PE) of the scaled CLr and the PBPK-derived CLr value, which is considered to be the “true” value, was calculated according to Eq. 7:

7 $$PE\left(\%\right)=\frac{{scaled\;CLr}_{obese}-PBPK\;{CLr}_{obese}}{{PBPK\;CLr}_{obese}}\times100\%$$

A PE value within ± 30% was regarded as accurate scaling. This strict threshold was chosen to minimize potential bias and uncertainty in the results. Systematic accuracy was defined as all hypothetical drugs within a defined category being scaled accurately.

Finally, the allometric exponent required to achieve the fully accurate scaling of CLr for each drug and each BMI category was calculated according to Eq. 8.

8 $$Exponent=\frac{In({CL}_{obese}/{CL}_{normal\;weight})}{In({BW}_{obese}/{BW}_{normal\;weight})}$$

## Results

### Obesity-related Changes in Renal Clearance

**Fig. 2:**
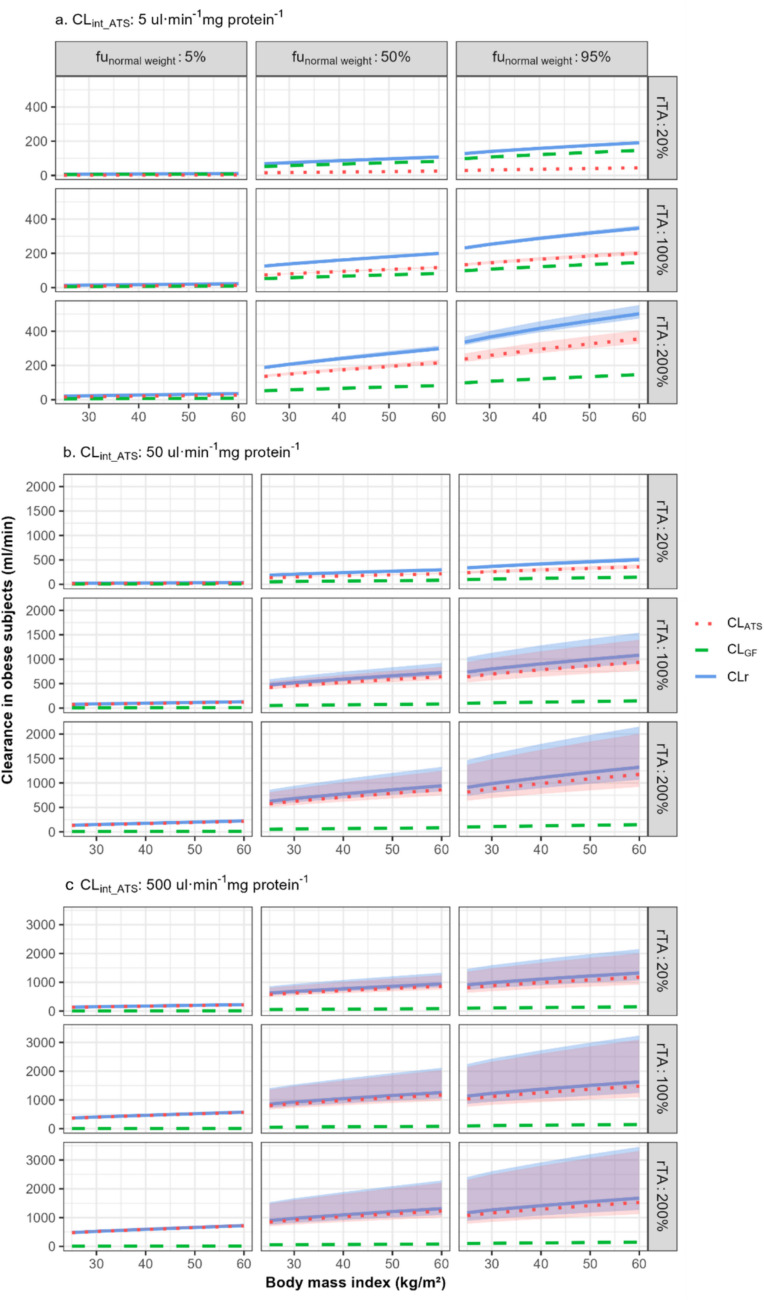
Total renal clearance (CLr, blue solid line) and the clearance through glomerular filtration (CL_GF_, green dashed line) and active tubular secretion (CL_ATS_, red dotted line) for typical overweight and obese subjects with body mass index (BMI) between 25 and 60 kg/m^2^ with low, normal, or high relative transporter activity (rTA) for hypothetical drugs that bind to human serum albumin (HSA). These drugs have low, intermediate, or high unbound fractions in normal-weight individuals (fu_normal weight_) and (**a**) low, (**b**) intermediate, or (**c**) high transporter-mediated intrinsic clearance values (CL_int_ATS_). Blue and red shaded areas represent the range of CLr and CL_ATS_, respectively, which results from differences in Kp (0.35, 1 or 4) impacting CL_ATS_. Note that the overlapped area is shaded as purple and that the scale of the y-axis varies between panels

Figure 2 illustrates how CL_GF_, CL_ATS,_ and total CLr differ between typical subjects with normal weight, overweight, and (morbid) obesity with varying rTA, for 27 representative drugs that bind to HSA, and that have different CL_int_ATS_ and fu_normal weight_ values. Results obtained for drugs that bind to AAG are shown in Supplementary Figure S1. The values of CL_GF_ and CL_ATS_ for these hypothetical drugs can be found in supplemental Table S1 and Table S2.

From these figures and tables, it can be seen that both CL_GF_ and CL_ATS_ increase with BMI, albeit to different extents depending on drug properties. For drugs binding to HSA, CL_GF_ and CL_ATS_ consistently increase with increasing BMI, regardless of whether fu is low or high. In contrast, for drugs binding to AAG, CL_GF_ and CL_ATS_ largely increase with BMI only when fu is intermediate or high (fu_normal weight_ ≥ 50%). When fu is low (e.g. fu_normal weight_ = 5%), CL_GF_ remains stable while CL_ATS_ only slightly increases with BMI. The observed differences are attributed to the decrease in HSA and the increase in AAG concentrations with rising BMI, as can be seen from Fig. 1. This leads to an increase in fu for HSA and a decrease in fu for AAG. Consequently, for drugs binding to HSA, CL_GF_, which depends on GFR and fu, rises with increasing BMI due to the combined effects of elevated GFR and increasing fu. In contrast, for AAG, the reduced fu counteracts the GFR increase, resulting in a relatively stable CL_GF_ with obesity. The influence of obesity-related changes in fu on CL_ATS_ is similar to its impact on CL_GF_, causing CL_ATS_ to increase with obesity for drug binding to HSA, while it has a decreasing influence on CL_ATS_ for drug binding to AAG.

In addition to fu, obesity-related changes in CL_ATS_ result from the interplay of other factors, including CL_int_ATS_, K_p_, rTA, kidney weight, renal blood flow, and GFR. As shown in Fig. 1, kidney weight, renal blood flow, and GFR all increase with rising BMI, partially explaining why obesity-induced changes in CL_ATS_ can potentially be more pronounced than changes in CL_GF_. Figure 2 and Figure S1 show that rTA can either amplify or dampen the impact of obesity-induced changes in the other variables that impact CL_ATS_, depending on the direction of change in rTA. It should be noted with these figures that rTA is unlikely to remain constant with BMI. It is more likely that differences in rTA become more extreme as BMI increases. Finally, these figures also show that as CL_int_ATS_ and fu_normal weight_ increase, the range of CL_ATS_ resulting from varying K_p_ values also expands, ultimately leading to a wider range in CLr.

Figure 2 and Figure S1 also illustrate the relative contribution of CL_GF_ and CL_ATS_ to total CLr, providing insights into whether changes in total CLr are predominantly driven by GF or ATS. In these figures, a line closer to the total CL line indicates a higher contribution from either GF or ATS to the overall renal clearance. These figures demonstrate that GF contributes significantly to total CLr only when CL_int_ATS_ is low or rTA is considerably reduced.

**Fig. 3:**
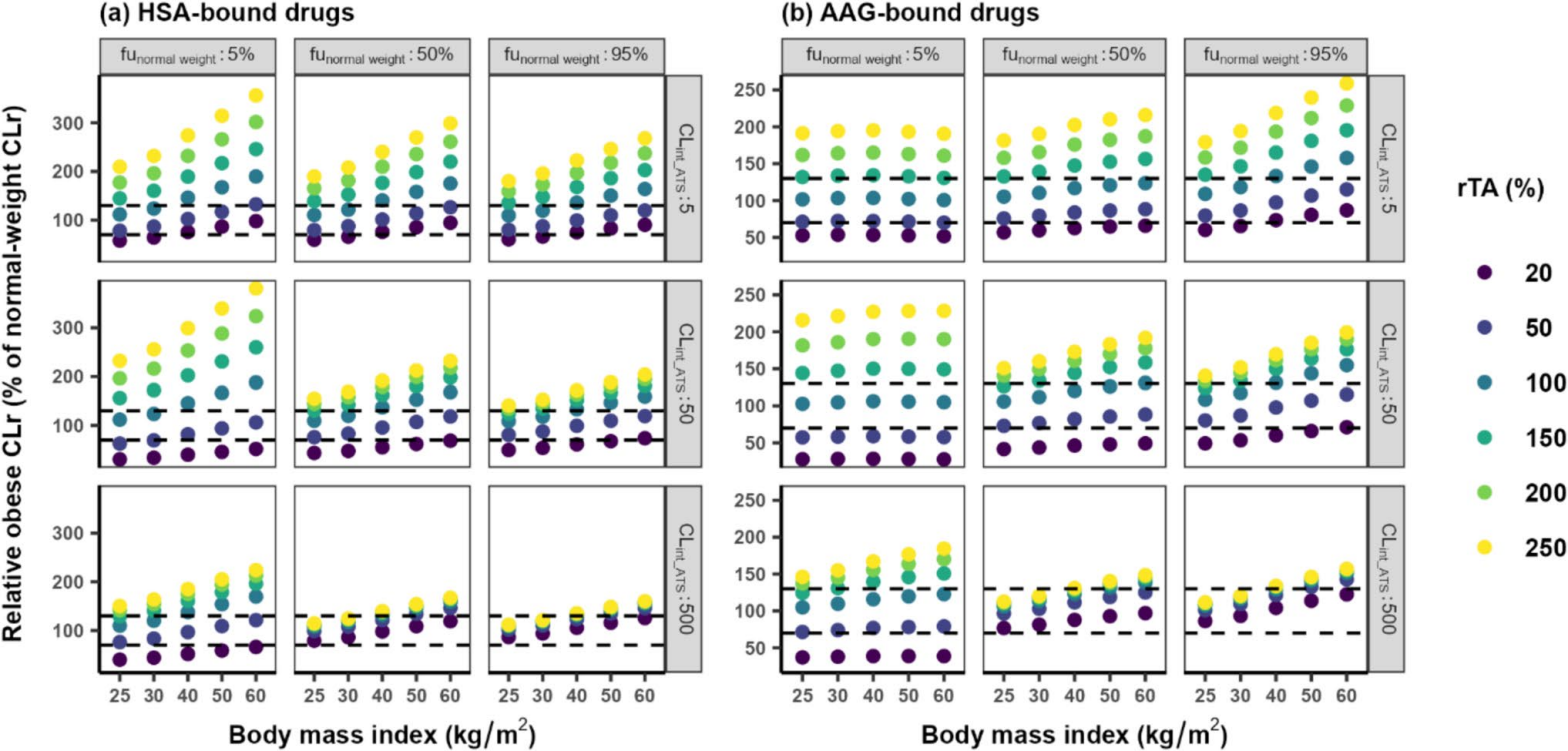
Relative total renal clearance (% of CLr for normal weight subjects) for typical overweight and obese subjects with body mass index (BMI) between 25 and 60 kg/m.^2^ with different relative transporter activity (rTA) for hypothetical drugs that bind to (**a**) human serum albumin (HSA) or (**b**) alpha1-acid glycoprotein (AAG). These drugs have low, intermediate, or high unbound fractions in normal-weight individuals (fu_normal weight_), low, intermediate or high transporter-mediated intrinsic clearance values (CL_int_ATS_), and a K_p_ value of 1. Note that the scale of the y-axis differs between (**a**) and (**b**). The dashed black lines represent the threshold of change in CLr within ± 30% (i.e. 70% ~ 130%)

The relative CLr in the obese compared to CLr in normal-weight individuals is illustrated in Fig. 3. This figure shows that when BMI is below 30 kg/m^2^ and relative transporter activity (rTA) remains unchanged, the CLr in obese subjects compared to normal-weight subjects exhibited a change generally confined within ± 30%, regardless of drug properties. In this case, the impact of obesity on CLr is considered negligible and flat dosing would be appropriate. Besides, this figure also indicates that for drugs with high protein binding (i.e., fu_normal weight_ = 5%), when the drug is highly bound to AAG, the change in relative CLr remains fairly stable across BMI values ranging from 25 to 60 kg/m^2^. Similarly, only small changes are identified for drugs with low protein binding (fu_normal weight_ = 95%) with high CL_int_ATS_ (500 ul·min^−1^ mg protein^−1^), binding to either HSA or AAG. Also for these drugs, flat dosing would be appropriate.

### Systematic Accuracy of CLr Scaling Methods

**Fig. 4:**
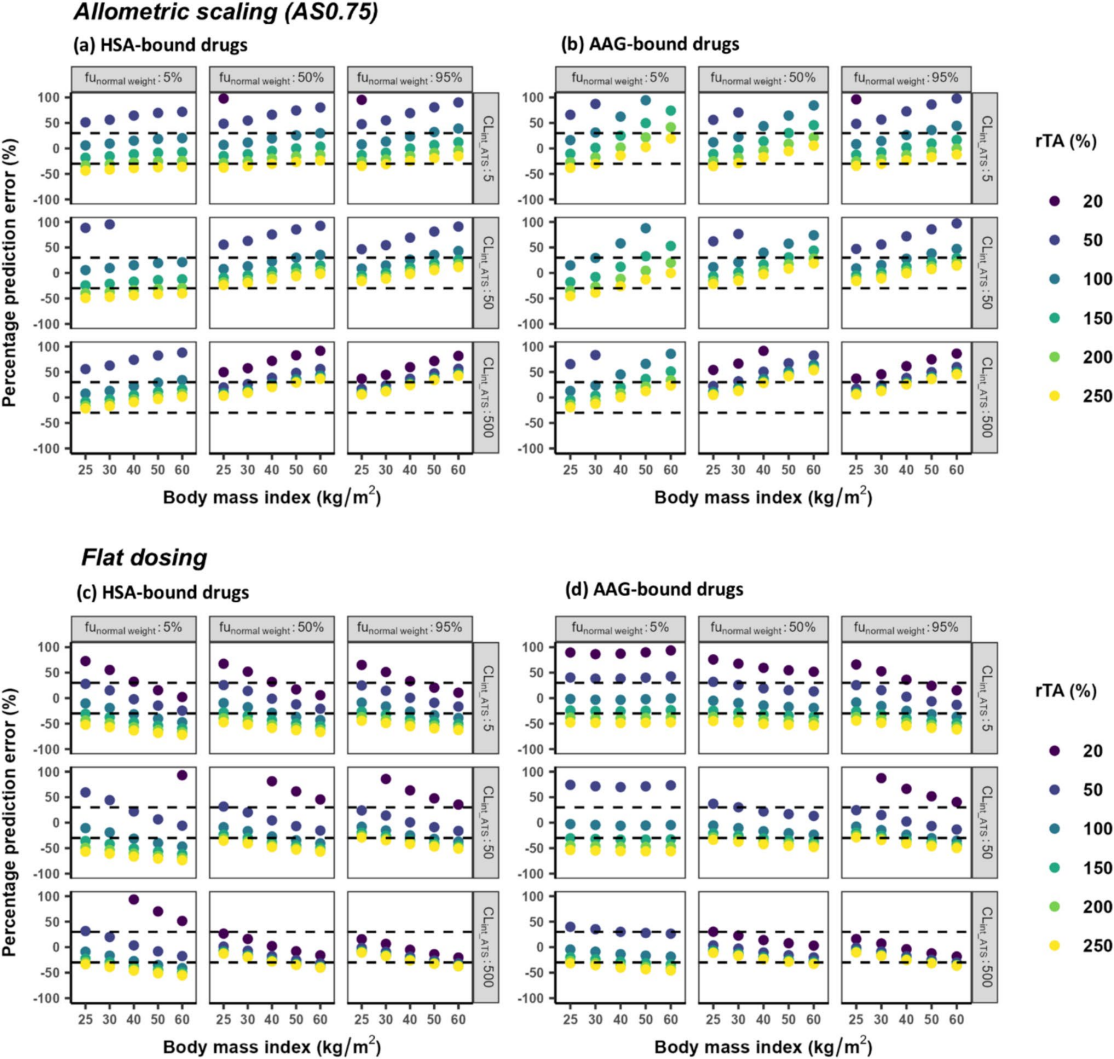
Percentage prediction error (PE) between renal clearance (CLr) values scaled using ***allometric scaling*** with an exponent of 0.75 (AS0.75, upper panel) or ***flat dosing*** with an exponent of 0 (bottom panel) and CLr obtained using the PBPK model for typical subjects with body mass index (BMI) between 25 and 60 kg/m^2^ and different relative transporter activity (rTA) for hypothetical drugs that bind to (**a, c**) human serum albumin (HSA) and (**b, d**) alpha1-acid glycoprotein (AAG). These drugs have low, intermediate, or high unbound fractions in normal-weight individuals (fu_normal weight_), low, intermediate or high transporter-mediated intrinsic clearance values (CL_int_ATS_), and a K_p_ value of 1. The black dotted lines represent the threshold of reasonably acceptable CLr prediction of ± 30%. Note that only changes in the range of ± 100% are shown

To support the *a priori* selection of dose scaling methods in a clinical setting, the PE values of CLr values scaled to obese individuals with an allometric equation with an exponent of 0 (assumed in flat dosing) and 0.75 are presented in Fig. 4. These plots indicate that in general, both AS0.75 and flat dosing are systematically accurate only for subjects with a BMI below 30 kg/m^2^, provided their transporter activity remains unchanged. At BMI > 30 kg/m^2^ or when transporter activity changes, changes in CLr become too variable to apply generalizable scaling rules, and systematic scaling accuracy can only be defined for specific scenarios taking subject and/or drug properties into account. For instance, AS0.75 is generally accurate for all investigated BMIs in scenarios in which CL_int_ATS_ is at or below 50 ul·min^−1^mg protein^−1^ and transporter activity increases by up to 150% for drugs binding to HSA or up to 250% for drugs binding to AAG. In scenarios in which transporter activity is reduced to 50%, flat dosing becomes the only accurate scaling method, especially in subjects with a BMI over 30 kg/m^2^ for drugs binding to HSA. For drugs binding to AAG, flat dosing is accurate as long as there is no change in transporter activity, irrespective of BMI or drug properties. Additionally, flat dosing remains accurate when fu_normal_weight_ exceeds 50% and CL_int_ATS_ reaches 500 ul·min^−1^mg protein^−1^, regardless of BMI, transporter activity changes, and drug-binding proteins. In all other scenarios, neither scaling method achieves systematically accurate scaling, meaning that it cannot be determined whether a scaling method is accurate based on BMI, fu, and CL_int_ATS_ alone and that PBPK-based approaches are required to ascertain accurate scaling.

To further increase our understanding of patterns in scaling accuracy, we also investigated for the various drugs and scenarios, which allometric exponent would be required to achieve fully accurate scaling of CLr. Table S3 and in Figure S2 show that the range of allometric exponents required for perfect scaling falls within −6.26 to 4.17, with the range decreasing as BMI increases. Notably, there is a strong correlation between the exponent and rTA. When rTA is 100%, the exponent remains relatively stable across the BMI range between 0.33 and 0.59 for drugs binding to HSA and between 0.0053 and 0.43 for drugs binding to AAG. The exponent tends to decrease with decreasing transporter activity and increase with increasing transporter activity.

## Discussion

In this study, a systematic analysis using a PBPK-based approach was used to quantify obesity-related changes in CLr, across a large number of hypothetical drugs eliminated via GF and various degrees of ATS. How drug and subject characteristics correlate with these changes was studied and the impact of obesity-induced changes in ATS on the total CLr for these drugs under various scenarios of change with obesity was explored. Moreover, this study also assessed the systematic accuracy of two commonly used weight-based scaling methods in predicting CLr.

This study delves into the physiological mechanisms underlying alterations in CLr among obese individuals. Given that the validity of this PBPK-based study relies on the accuracy of the underlying physiological parameters and assumption, its findings are compared with results from clinical studies. Studies of daptomycin [20, 21] and cefazolin [22–24], for instance, reported an unaltered CLr in subjects with varying degrees of obesity compared to normal-weight subjects, suggesting that a flat dose or a weight-independent fixed dose is appropriate when aiming for similar plasma concentrations. Daptomycin and cefazolin, both exhibit high plasma protein binding (fu_normal weight_ < 20%), primarily undergo GF with very limited involvement of ATS [25–27]. Therefore, these findings can be explained by our results shown in Fig. 2 and Table S1, indicating limited changes of CLr for drugs eliminated through GF with a low fu value, irrespective of the degree of obesity. When comparing our results to literature, it should be noted that our results are based on absolute CLr values, limiting direct comparisons of findings on CLr expressed per kg bodyweight.

For drugs for which both GF and ATS contribute considerably to CLr, the patterns in the changes in CLr are more complex and the impact of transporter activity needs to be considered as well. For instance, a study of meropenem reported that the CLr in obese subjects was not significantly different from that of normal-weight subjects [28]. Meropenem is barely bound to serum proteins and is a substrate of organic anion transporter (OAT) 1 and 3 [29]. The findings from this study suggest that for a drug with low protein binding, an increase in CLr would be expected unless rTA is low. Therefore, the unchanged CLr of meropenem could potentially be attributed to a reduction in OAT activity in subjects with obesity. Conversely, in the case of drugs such as metformin [30], gentamicin [31, 32] and vancomycin [3, 33, 34], which exhibit lower serum protein binding, their increased CLr is likely due to the increased activity of organic cation transporter (OCT) 1 or 2 involved in their ATS [17, 35, 36].

In subjects with overweight or obesity, both CL_GF_ and CL_ATS_ were found to increase with BMI, though the extent varied considerably depending on drug properties. In addition to BMI, the change in CL_GF_ primarily depended on the changes in fu, while changes in CL_ATS_ were influenced by multiple parameters, including CL_int_ATS_, fu_normal weight_, rTA, K_p_, kidney weight, renal blood flow, and GFR. Notably, in this study, the transporter activity in individuals with overweight and obesity was evaluated relative to normal-weight subjects. When transporter activity decreased due to obesity (i.e., rTA below 100%), the absolute value of CL_ATS_ or CLr may still increase with increasing BMI, but could remain lower than those in normal-weight subject.

This study also evaluated the systematic accuracy of two commonly used scaling methods in predicting CLr for obese individuals, which is clinically relevant to guide appropriate dose scaling methods, particularly in those cases where results from clinical studies are lacking or in case of novel drugs. When scaling is systematically accurate, the PE of all scaled CLr values is less than ± 30%, which minimizes potential bias and uncertainty in the results. The absence of systematic accuracy does not mean that scaling is inaccurate for all drugs, it merely indicates that it cannot be known *a priori* whether CLr scaling for a drug will be accurate without taking additional information into account. We found that when subjects had a BMI below 30 kg/m^2^ and unaltered transporter activity, both AS0.75 and flat dosing methods were systematically accurate for any drugs. Under any other circumstance, details regarding the drug elimination pathway (i.e., degree of ATS), fu_normal weight_, CL_int_ATS_, rTA, and/or the degree of obesity are required to be known for the *a priori* selection of an appropriate scaling method with the help of Fig. 4. In cases when systematic accuracy is not achieved, PBPK-based approaches are essential for accurate CLr predictions in individuals with obesity. For instance, our results show that when transporter activity decreases below 50%, neither scaling method could achieve systematically accurate scaling. This is because, as illustrated in Figure S2, the exponent required for accurate scaling becomes negative in this scenario. PBPK modeling is the only appropriate method for predicting obesity-related CLr changes in such cases.

The use of a PBPK approach enabled us to predict drug clearance by considering various drug properties, subject characteristics, and different degrees of obesity, in the absence of clinical data on individual drugs. Previously, we applied this approach to hepatically cleared drugs in obesity [37] and for hepatically and renally cleared drugs in the pediatric population [6–8]. Using this approach, rather than studying a limited number of existing drugs, we generated a total of 11,520 hypothetical drugs to cover the entire parameter space with realistic ranges for each parameter, thereby deriving generalizable guidance on required dose adjustments in those cases where no evidence from clinical studies is available. Similar to the previous studies, we cannot entirely exclude the possibility that some generated drug property combinations are uncommon or unrealistic. Nonetheless, our findings will likely cover all currently existing drugs as well as small molecule drugs that are still to be developed in the future. As mentioned before, for rTA, unrealistic combinations of subject properties were likely generated, for instance, when BMI is 25 kg/m^2^, a 250% obesity-related increase in rTA is unlikely, but as it is unknown what ranges in rTA are realistic for the different BMI categories, our results cover a wide range of possible scenarios. We emphasize that when clinical data become available for existing drugs, population PK approaches are more suitable to quantify inter-individual variability and covariate effects, which are required to derive dedicated dose adjustments for each individual drug.

Our results show that obesity induced changes in rTA have a significant impact on clearance scaling to the obese, which highlights the need to quantify this parameter for different renal transporters to achieve accurate predictions in obese. It is also worth mentioning that the overweight and obese subjects included in this PBPK study were defined based on BMI. The reason for this is that the only available literature reporting subject-specific parameters for overweight and obese subjects is based on BMI rather than bodyweight. If future studies demonstrate better correlation for some of the parameters with bodyweight or other descriptors, this can be easily adapted in the current workflow. Moreover, this PBPK model incorporates renal clearance pathways for GF and ATS, while intentionally excluding reabsorption and renal metabolism as renal elimination routes, to reduce the complexity of the findings. The extrapolation potential of our findings to drugs that are also cleared through other routes becomes increasingly uncertain as the contribution of the other routes increases. Renal metabolism of drugs mainly undergoing renal clearance is however likely very small since kidney isoenzymes are also found in the liver. Nonetheless, exploring the impact of reabsorption, along with its dependence on physiological factors like ionization and pH, or renal metabolism could be investigated using similar approaches in future analyses. Finally, we defined the range of relative changes in obesity-induced transporter activity (rTA) according to the existing literature involving obese mice [14]. This is due to the fact that there is limited quantitative data on the change in transporter activity in obese humans. Upon the availability of quantitative data regarding obesity-induced changes in specific transporter activities, our findings can serve as a valuable tool for predicting corresponding alterations in CLr.

## Conclusion

In this study, we found that obesity-induced changes in fu, CL_int_ATS_, and rTA primarily drive the obesity-related changes in CLr. How fu changes with BMI depends on the plasma protein the drug binds to. For drugs binding to HSA, CLr consistently increases with BMI regardless of fu changes, whereas for drugs binding to AAG, CLr remains relatively stable as the direction of the change in fu negates the impact of increased CL driven by other variables. Based on the obesity-related changes in CLr that were quantified with PBPK modeling, we could conclude that no single clearance scaling method can be systematically accurate for drugs cleared through GF and various degrees of ATS when BMI is higher than 30 kg/m^2^ or when transporter activity is impacted by obesity. The impact of rTA on obesity-related changes in clearance and the current lack of data on rTA prevents *a priori* selection of a unique appropriate scaling method. and even limits PBPK model predictiveness for these drugs. This highlights the need to gather information on rTA to allow for accurate prediction of renal clearance in obese. In the meantime, the results of this study could inform the selection of a set of appropriate scaling methods for subsets of drugs, based on fu, CL_int_ATS_, drug binding protein, and range of probable rTA to define a range of clearance in obese patients.

## Supplementary Information

Below is the link to the electronic supplementary material.

Supplementary file1 (PDF 1013 KB)

## Data Availability

The datasets generated during and/or analysed during the current study are available from the corresponding author on reasonable request.
