# Supplementary material for: The Impact of Drug Properties and Severity of Obesity on Renal Drug Clearance Through Glomerular Filtration and Active Tubular Secretion: A Systematic Analysis Using PBPK Modeling
Source: Pharm Res. 2025 Jun 27;42(7):1079–88. doi: 10.1007/s11095-025-03885-5 (PMC12304071; doi:10.1007/s11095-025-03885-5)
Supplement: Supplementary file 1 — Supplementary file1 (PDF 1013 KB) [file 11095_2025_3885_MOESM1_ESM.pdf]

Supplemental materials

The impact of drug properties and severity of obesity on renal drug clearance through glomerular filtration and active tubular secretion: a systematic analysis using PBPK modeling

Authors

Tan Zhang (1,2), Elisa A.M. Calvier (3), Elke H.J. Krekels (1,2)*, Catherijne A.J. Knibbe*[†] (1,4)

Affiliations

(1) Division of Systems Pharmacology and Pharmacy, Leiden Academic Centre for Drug Research, Leiden University, Leiden, The Netherlands

(2) Certara Inc, Princeton, NJ, USA

(3) Pharmacokinetics-Dynamics and Metabolism, Translational Medicine and Early Development, Sanofi R&D, Montpellier, France

(4) Department of Clinical Pharmacy, St. Antonius Hospital, Nieuwegein, The Netherlands

*These two authors contributed equally

Corresponding author

Catherijne A.J. Knibbe[†]

Department of Clinical Pharmacy, St. Antonius Hospital, 3430 EM Nieuwegein, The Netherlands

Telephone number: 088 320 7201

E-mail address: c.knibbe@antoniusziekenhuis.nl

Supplemental equations

1. Equations and the corresponding values of subject-specific parameters for the typical individuals implemented in the PBPK-based simulation workflow

System-specific parameters	Equations from Berton *et.al* [1]	Demographic values					
		non-obese	obese				
Body mass index (BMI, kg/m^2)	--	20	25	30	40	50	60
Height (HT, cm)	--	172	172	172	172	172	172
Body weight (BW, kg)	$\mathrm{BMI} \times (\mathrm{HT}/100)^2$	59.2	74	88.8	118	148	178
Fat mass (FM, kg)	$[(30.86 - 3.09 \times \mathrm{Sex} - 0.13 \times \mathrm{HT} + 0.14 \times \mathrm{BW}) - \mathrm{BMI}] \times (\mathrm{HT}/100)^2$	14.1	22.7	31.4	48.8	66.1	83.4
Cardiac output (CO, L/min)	$\mathrm{Heart\ rate} \times (43.13 + 12.96 \times \mathrm{Blood\ weight}) \times (0.65 - 0.0018 \times \mathrm{BMI})/1000$	5.86	6.29	6.81	7.59	8.31	8.96
Kidney blood flow (Qr, L/min)	$(20.57 - 1.76 \times \mathrm{Sex}) \times \mathrm{CO}/100$	1.15	1.24	1.34	1.49	1.64	1.76
Human serum albumin (HSA, g/L)	$47.58 - 0.16 \times \mathrm{BMI}$	44.4	43.6	42.8	41.2	40.0	38.0
Alpha-1 acid glycoprotein (AAG, g/L)	$0.48 + 0.012 \times \mathrm{BMI}$	0.72	0.78	0.84	0.96	1.08	1.20
Hematocrit	$0.45 - 0.04 \times \mathrm{Sex}$	0.43	0.43	0.43	0.43	0.43	0.43
Kidney weight (KW, kg)	$-0.11 - 0.048 \times \mathrm{Sex} + 0.003 \times \mathrm{Age} + 0.18 \times \mathrm{Body\ surface\ area}$	0.31	0.35	0.38	0.44	0.48	0.53
Glomerular filtration rate (GFR, ml/min)	$13.02 - 0.51 \times \mathrm{Age} + 0.93 \times (\mathrm{FM}/\mathrm{BW}) + 93.56 \times \mathrm{Qr}/1000$	95.7	104	113	128	141	153

Note: when calculating values that depend on sex, the average value for both sexes was used. Age was set as 50 years when required for parameter calculations.

2. Equations to calculate the drug- and system-specific parameters for the typical individuals implemented in the PBPK-based simulation workflow

$$fu_{obese} = \frac{1}{1+\frac{(1-fu_{normal\ weight}) \times PP_{obese}}{fu_{normal\ weight} \times PP_{normal\ weight}}} \quad \text{(Eq. 1)}$$

$$CL_{int\_sec} = CL_{int\_ATS} \times PTCPGK \times Kidney\ weight \times rTA \quad \text{(Eq. 2)}$$

$$B{:}P = 1 + (Hematocrit * (fu * K_p - 1)) \quad \text{(Eq. 3)}$$

In these equations, values for $fu_{normal\ weight}$, CL_{int_ATS}, and K_p are taken from normal-weight subjects as defined in the main text. PP is the concentration of plasma protein, which was assumed to be either human serum albumin (HSA) or alpha-1 acid glycoprotein (AAG). The number of proximal tubule cells per gram kidney (PTCPGK) was assumed to be 99.4, according to the SimCYP Simulator® (version 22), and is assumed to remain unaltered across varying BMI. Obesity-induced changes in transporter activity are integrated as fractions relative to transporter-mediated active clearance in normal-weight individuals (rTA).

Supplemental tables

Table S1. Clearance through glomerular filtration for the investigated body mass index (BMI) for hypothetical drugs categorized per drug binding protein with low to high unbound drug fraction in normal weight subjects ($fu_{normal\ weight}$)

	**BMI (kg/m²)**	**Clearance through glomerular filtration for drugs with varying $fu_{normal\ weight}$**		
		**5%**	**50%**	**95%**
**HSA-bound drugs**	25	5.27 ml/min	52.31 ml/min	98.58 ml/min
	30	5.87 ml/min	57.69 ml/min	107.83 ml/min
	40	6.86 ml/min	66.25 ml/min	121.77 ml/min
	50	7.86 ml/min	74.55 ml/min	134.71 ml/min
	60	8.87 ml/min	82.46 ml/min	146.42 ml/min
**AAG-bound drugs**	25	4.8 ml/min	49.77 ml/min	98.09 ml/min
	30	4.89 ml/min	52.29 ml/min	106.75 ml/min
	40	4.85 ml/min	54.74 ml/min	119.34 ml/min
	50	4.78 ml/min	56.41 ml/min	130.71 ml/min
	60	4.68 ml/min	57.38 ml/min	140.68 ml/min

HSA, human serum albumin, AAG, alpha-1 acid glycoprotein

Table S2. Clearance through active tubular secretion for the investigated body mass index (BMI) for hypothetical drugs categorized per drug binding protein with low to high unbound drug fraction in normal-weight subjects ($fu_{normal\ weight}$) and transporter-mediated intrinsic clearance values (CL_{int_ATS}). These drugs have K_p values of 0.35, 1, and 4. Note that obesity-induced changes in transporter activity are not considered in this table, and that total clearance is the sum of clearance through both active tubular secretion and glomerular filtration.

	BMI (kg/m^2)	CL_{int_ATS} ($ul \cdot min^{-1} mg\ protein^{-1}$)	Clearance through active tubular secretion for drugs with varying $fu_{normal\ weight}$		
			**5%**	**50%**	**95%**
**HSA-bound drugs**	25	5	8 [7.99, 8.05] ml/min	76.67 [72.28, 79.58] ml/min	143.16 [127.34, 149.94] ml/min
		50	73.06 [71.97, 77.05] ml/min	549.8 [382.95, 744.97] ml/min	971.74 [527.03, 1400.99] ml/min
		500	391.35 [362.02, 541.51] ml/min	1438.67 [671.63, 4545.7] ml/min	2313.58 [768.11, 8457.57] ml/min
	30	5	8.84 [8.83, 8.9] ml/min	84.06 [79.17, 87.28] ml/min	155.68 [138.4, 163.09] ml/min
		50	80.66 [79.41, 85.17] ml/min	601.45 [417.22, 816.73] ml/min	1055.18 [571.27, 1523.38] ml/min
		500	428.46 [395.55, 596.64] ml/min	1567.53 [728.08, 4974.21] ml/min	2506.29 [831.28, 9180.75] ml/min
	40	5	10.5 [10.48, 10.57] ml/min	97.96 [91.99, 101.85] ml/min	178.42 [158.1, 187.16] ml/min
		50	95.24 [93.64, 100.97] ml/min	694.1 [475.47, 951.24] ml/min	1198.53 [642.9, 1744.9] ml/min
		500	493.26 [453.09, 698.63] ml/min	1776.96 [815.39, 5730.58] ml/min	2806.61 [927.24, 10404.15] ml/min
	50	5	12.06 [12.03, 12.14] ml/min	110.37 [103.43, 114.84] ml/min	197.67 [174.9, 207.47] ml/min
		50	108.92 [106.95, 115.85] ml/min	778.09 [528.12, 1071.71] ml/min	1322.93 [706.67, 1932.72] ml/min
		500	553.64 [506.14, 795.56] ml/min	1973.79 [896.04, 6426.37] ml/min	3080.1 [1015.4, 11474.47] ml/min
	60	5	13.69 [13.66, 13.79] ml/min	122.82 [114.83, 127.9] ml/min	216.2 [190.97, 227.08] ml/min

		50	123.1 [120.69, 131.42] ml/min	860.79 [578.44, 1192.44] ml/min	1440.52 [765.71, 2113.41] ml/min
		500	613 [557.49, 894.86] ml/min	2161.01 [970.12, 7110.4] ml/min	3330.65 [1095.38, 12481.3] ml/min
**AAG-bound drugs**	25	5	7.29 [7.28, 7.33] ml/min	73 [69.06, 75.71] ml/min	142.44 [126.78, 149.19] ml/min
		50	67.04 [66.19, 70.3] ml/min	526.12 [372.73, 708.88] ml/min	967.26 [525.75, 1393.93] ml/min
		500	371.19 [346.59, 499.33] ml/min	1389.8 [665.27, 4330.43] ml/min	2304.24 [767.18, 8415.52] ml/min
	30	5	7.39 [7.38, 7.42] ml/min	76.32 [72.37, 79.11] ml/min	154.13 [137.18, 161.45] ml/min
		50	68.22 [67.45, 71.26] ml/min	551.58 [395.52, 740.64] ml/min	1045.47 [568.52, 1508.11] ml/min
		500	386.41 [363.07, 509.69] ml/min	1464.8 [714.63, 4521.12] ml/min	2486.14 [829.26, 9089.84] ml/min
	40	5	7.45 [7.44, 7.48] ml/min	81.2 [77.26, 84.16] ml/min	174.9 [155.35, 183.43] ml/min
		50	69.24 [68.6, 71.92] ml/min	587.03 [428.78, 786.67] ml/min	1176.7 [636.8, 1710.19] ml/min
		500	405.22 [384.39, 518.46] ml/min	1559.57 [786.76, 4761.07] ml/min	2761.9 [922.79, 10199.82] ml/min
	50	5	7.37 [7.36, 7.39] ml/min	83.93 [80.15, 86.92] ml/min	191.88 [170.37, 201.32] ml/min
		50	68.85 [68.34, 71.17] ml/min	609.53 [453.96, 812.12] ml/min	1287.1 [696.7, 1875.53] ml/min
		500	416.78 [398.41, 518.92] ml/min	1633.57 [850.77, 4903.67] ml/min	3007.09 [1008.14, 11139.21] ml/min
	60	5	7.27 [7.27, 7.3] ml/min	86.02 [82.41, 89.03] ml/min	207.83 [184.44, 218.18] ml/min
		50	68.32 [67.88, 70.34] ml/min	627.07 [475.02, 831.39] ml/min	1388.94 [751.46, 2030.7] ml/min
		500	425.24 [408.95, 517.82] ml/min	1692.53 [907.27, 5003.7] ml/min	3226.26 [1085.02, 11999.04] ml/min

HSA, human serum albumin, AAG, alpha-1 acid glycoprotein

Table S3. Allometric exponent required for accurate scaling for the investigated body mass index (BMI) for hypothetical drugs categorized per drug binding protein with low to high unbound drug fraction in normal-weight subjects ($fu_{normal\ weight}$) and transporter-mediated intrinsic clearance values (CL_{int_ATS}). Note that obesity-induced changes in transporter activity are not considered in this table.

	**BMI (kg/m²)**	**CL_{int_ATS} (ul·min⁻¹mg protein⁻¹)**	**Allometric exponent required for accurate scaling for drugs with varying $fu_{normal\ weight}$**		
			5%	50%	95%
**HSA-bound drugs**	25	5	0.49 [0.49- 0.49]	0.45 [0.45- 0.46]	0.42 [0.41- 0.42]
		50	0.51 [0.51- 0.52]	0.44 [0.40- 0.48]	0.41 [0.37- 0.45]
		500	0.43 [0.42- 0.48]	0.37 [0.34- 0.44]	0.34 [0.33- 0.40]
	30	5	0.53 [0.52- 0.53]	0.48 [0.48- 0.49]	0.44 [0.44- 0.45]
		50	0.53 [0.52- 0.54]	0.47 [0.44- 0.49]	0.43 [0.40- 0.45]
		500	0.46 [0.45- 0.50]	0.42 [0.39- 0.46]	0.39 [0.38- 0.42]
	40	5	0.55 [0.55- 0.55]	0.49 [0.49- 0.50]	0.45 [0.44- 0.45]
		50	0.55 [0.54- 0.56]	0.48 [0.45- 0.51]	0.43 [0.41- 0.46]
		500	0.47 [0.46- 0.52]	0.42 [0.39- 0.47]	0.39 [0.38- 0.43]
	50	5	0.56 [0.56- 0.56]	0.50 [0.50- 0.51]	0.45 [0.44- 0.45]
		50	0.56 [0.56- 0.57]	0.49 [0.45- 0.51]	0.44 [0.41- 0.46]
		500	0.48 [0.47- 0.54]	0.44 [0.40- 0.48]	0.40 [0.39- 0.43]
	60	5	0.58 [0.58- 0.58]	0.52 [0.51- 0.52]	0.45 [0.45- 0.46]
		50	0.58 [0.57- 0.59]	0.50 [0.46- 0.53]	0.44 [0.42- 0.46]
		500	0.50 [0.48- 0.55]	0.45 [0.41- 0.50]	0.40 [0.39- 0.44]
**AAG-bound drugs**	25	5	0.07 [0.07- 0.07]	0.23 [0.23- 0.24]	0.39 [0.38- 0.40]
		50	0.12 [0.11- 0.13]	0.25 [0.25- 0.27]	0.39 [0.35- 0.42]
		500	0.19 [0.11- 0.22]	0.22 [0.20- 0.28]	0.32 [0.32- 0.38]
	30	5	0.08 [0.08- 0.08]	0.24 [0.24- 0.25]	0.42 [0.41- 0.42]
		50	0.11 [0.09- 0.12]	0.25 [0.25- 0.29]	0.41 [0.39- 0.43]

	500	0.20 [0.11- 0.23]	0.25 [0.22- 0.33]	0.37 [0.36- 0.40]
	5	0.05 [0.05- 0.05]	0.22 [0.22- 0.23]	0.42 [0.41- 0.42]
40	50	0.08 [0.07- 0.09]	0.24 [0.23- 0.28]	0.41 [0.39- 0.43]
	500	0.19 [0.09- 0.22]	0.23 [0.20- 0.33]	0.37 [0.36- 0.40]
	5	0.02 [0.02- 0.02]	0.20 [0.20- 0.21]	0.42 [0.41- 0.42]
50	50	0.06 [0.04- 0.06]	0.22 [0.21- 0.27]	0.41 [0.39- 0.43]
	500	0.17 [0.07- 0.20]	0.23 [0.19- 0.33]	0.37 [0.37- 0.40]
	5	0.00 [0.0053- 0.01]	0.19 [0.19- 0.20]	0.42 [0.41- 0.42]
60	50	0.04 [0.02- 0.05]	0.21 [0.20- 0.27]	0.41 [0.40- 0.43]
	500	0.16 [0.06- 0.19]	0.22 [0.17- 0.33]	0.38 [0.37- 0.40]

HSA, human serum albumin, AAG, alpha-1 acid glycoprotein

Supplemental figures

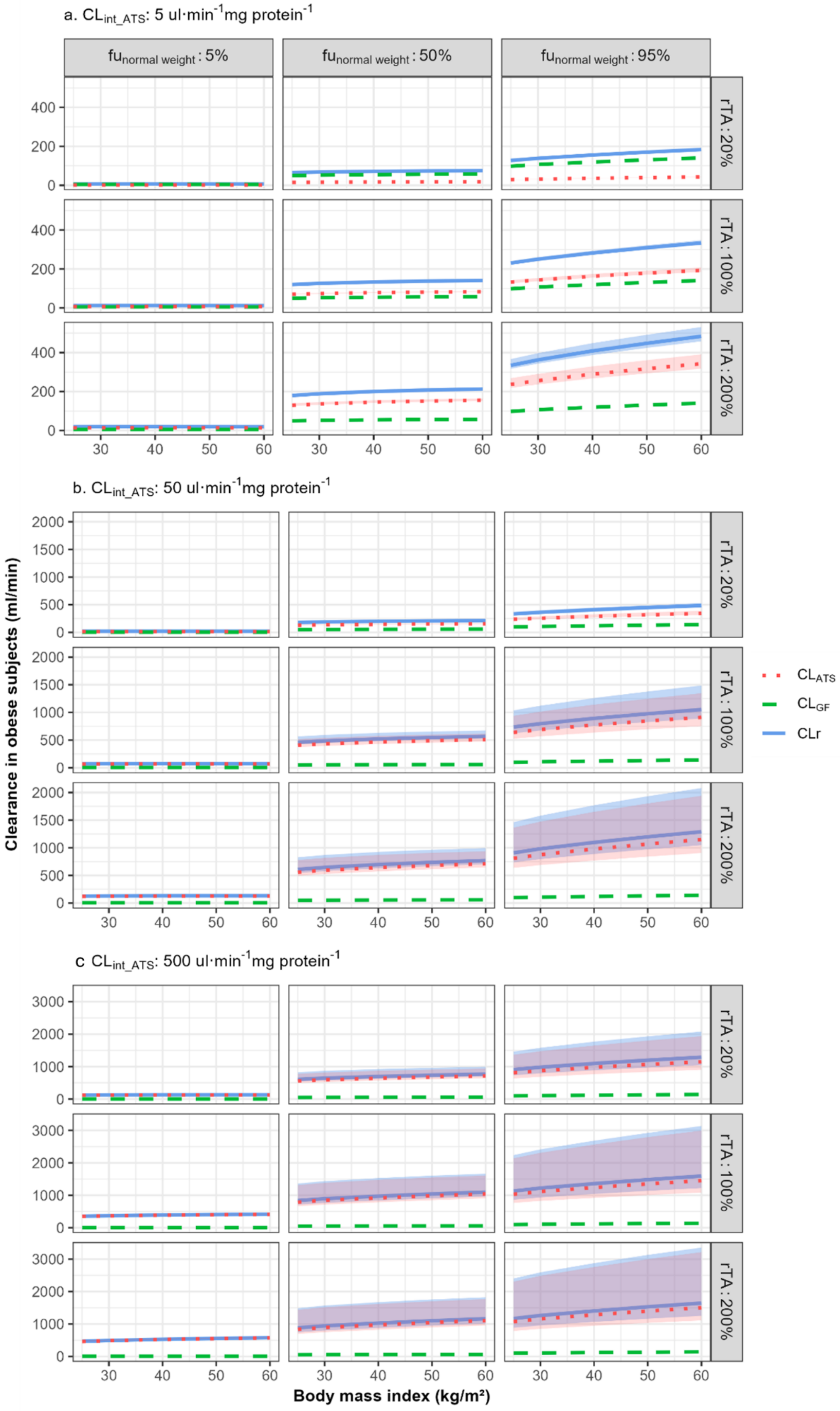

Fig. S1 Total renal clearance (CLr, blue solid line) and the clearance through glomerular filtration (CL_{GF}, green dashed line) and active tubular secretion (CL_{ATS}, red dotted line) for typical overweight and obese subjects with body mass index (BMI) between 25 and 60 kg/m^2 with low, normal or high relative transporter activity (rTA) for hypothetical drugs that bind to alpha-1 acid glycoprotein (AAG). These drugs have low, intermediate, or high unbound fractions in normal-weight individuals ($fu_{normal\ weight}$) and low, intermediate, or high transporter-mediated intrinsic clearance values (CL_{int_ATS}). Blue and red shaded areas represent the range of CLr and CL_{ATS}, respectively, which results from differences in Kp (0.35, 1 or 4) impacting CL_{ATS}. Note that the overlapped area is shaded as purple and that the scale of the y-axis varies between panels.

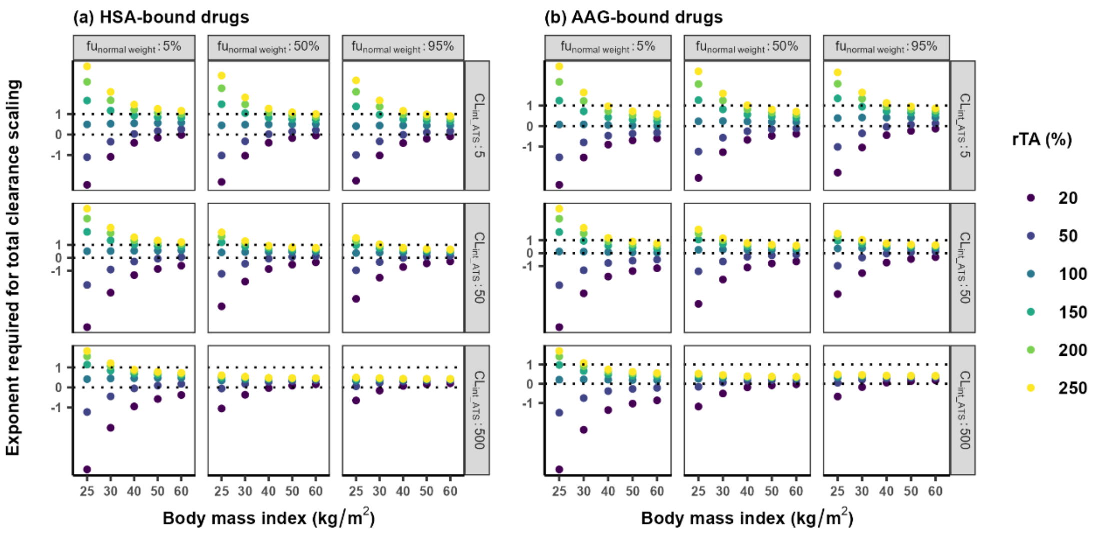

Fig. S2 Exponent required for total clearance scaling for typical overweight and obese subjects with body mass index (BMI) between 25 and 60 kg/m^2 with low, intermediate, or high relative transporter activity (rTA) for hypothetical drugs that bind to (**a**) human serum albumin (HSA) and (**b**) alpha1-acid glycoprotein (AAG). These drugs have low, intermediate, or high unbound fractions in normal-weight individuals ($fu_{normal\ weight}$), low, intermediate, or high transporter-mediated intrinsic clearance values (CL_{int_ATS}), and a K_p value of 1.

References

1. Berton M, Bettonte S, Stader F, Battegay M, Marzolini C. Repository Describing the Anatomical, Physiological, and Biological Changes in an Obese Population to Inform Physiologically Based Pharmacokinetic Models. *Clin Pharmacokinet*. 2022;61(9):1251-1270. doi:10.1007/s40262-022-01132-3
